# The Equipment of Emergency Hospitals

**Published:** 1914-08-29

**Authors:** 


### The Equipment of Emergency Hospitals..

### THE PRICE OF HOSPITAL BEDSTEADS.

We are informed by Messrs. J. Nesbit-Evans and Co. that the price at which they supplied the Admiralty during the week ending August 15 with a quantity of their special balcony hospital bedsteads from stock was 44s. per bedstead, and on August 21 they were still adhering to that price, although in two or three days' time they expected to have to increase the prices of bedsteads owing to the increased cost of materials. Messrs. J. Nesbit-Evans and Co. state they have not been guilty of increasing the price of their hospital bedsteads, as so freely mentioned in the press. In addition to the special balcony bedsteads referred to, of which they have about 300 to 400 ready for despatch, they have also 270 of a cheaper kind of hospital bed, having socket at head to receive head pole, but this bedstead has smaller wheels, and is similar to their No. 1,009 pattern. The price of this bedstead, which is quite a modern ordinary hospital bedstead, is 33s. 6d. each, carriage paid, size 6 ft. 6 in. by 3 ft.

### THE "JACKSON" PATENT WATER BOILER.

Beds and bedding are not the only or the first things required for hospital equipment. The question of an immediate and constant supply of boiling water requires careful consideration by all those responsible for purchasing the equipment for emergency hospitals. The "Jackson" patent water boiler was first designed for the use of caterers, but on account of its utility it has been adopted by hospitals and public institutions of many kinds where boiling water is required continuously, night or day. The "Jackson" is so designed that no impurities can affect the water, and the machine itself can be easily fixed by any skilled plumber wherever there is a gas and water supply. It is connected direct to the mains, and the water is boiled as it travels through the machine to the draw-off tap. All the waterways are tinned to ensure purity. The gas consumption is remarkably low, for whereas to boil a gallon of water in a kettle, urn, or similar vessel, eight to nine feet of gas are consumed, it is calculated that the "Jackson" provides a gallon of boiling water for every two and a-half cubic feet of gas. It is manufactured by Jackson Boilers, Ltd., 91 Leicester Place, Leeds, from whom specifications can be obtained.

### DINNER WAGONS.

In the Institutional Workers' Supplement of The Hospital of August 15 an important article by an officer of the Birmingham Union Infirmary, Dudley Road, appeared on the subject of dinner wagons. The specifications which were insisted on are of special importance now that emergency hospitals are being prepared and their equipment organised by many people without much experience of hospital management. An illustration of Messrs. Alldays and Onions' dinner wagons accompanied the article, and this type may be commended especially to those who are busy converting large halls and public buildings to hospital purposes, or undertaking the equipment of hospital camps. In both these cases meals have to be served in large wards, and to be kept hot often for a long time after leaving an improvised kitchen. The problem of serving hot meals is a great one even in hospitals specially designed, and its difficulty will be greatly enhanced in many emergency hospitals unless special precautions, such as properly designed dinner wagons, are secured. How valuable Messrs. Alldays and Onions' are may be judged from the following passage in the article: "When the tins were being tested," wrote the institutional officer alluded to, "I accompanied a wagon which served out soup to the wards in a corridor one-fifth of a mile in length, and when the work was finished tasted what was left and found the soup so hot that I had no wish to repeat the experiment." All equipping emergency hospitals will do well to bear in mind the efficiency of this system.

### THE FLOORS OF EMERGENCY HOSPITALS.

Those who are organising emergency hospitals often have to arrange many things which are afterwards submitted to and not always passed by expert medical opinion, and their difficulties are often increased by the gifts in kind which they receive. Beds, blankets, clothes: these everyone remembers, but in adapted buildings where carpets have to be removed and scrupulous cleanliness enforced the floors are one of the first things which require attention—ward stores of all kinds are apt to arrive before this essential has been dealt with. There is hardly a wooden floor, however, which will not be made as antiseptic as polish can make it, when not designed for hospital purposes, when the Judge Brand Company's (Gateshead) floor polishes are used. Easy to apply, requiring no elaborate apparatus, and inexpensive, they can be cordially recommended to those who are converting private houses and public buildings into wards. Walls, floors, and windows are the prime things to get into order when private houses are being adapted to hospital needs. Mere furniture and equipment can be altered or imported; not so the ceilings and floors. It is therefore essential at this time that the latter should be particularly examined and scoured. But this is not sufficient; they must be given a good smooth surface to be easily cleaned and stand hard wear. This purpose Judge Brand Company's floor polishes are excellently designed to secure. No. 2 concentrated is excellent. The company have placed on the market two varieties—Judge Antiseptic Floor Polishes, Numbers 1 and 2, concentrated, specially adapted for hospital floors, and now employed, it is understood, in several of our military hospitals. The concentrated is sold at 1s. per lb., and in 7-lb. and 14-lb. tins, while there is also a fluid polish, price 5s. 6d. per gallon. In addition, of course, specially designed polishing brushes and floor stains can be obtained from the same firm.

### DESTRUCTION OF DRESSINGS.

At the present time, with the prospect of wounded and possibly also of sick arriving in large numbers from the Continent, the question of hospital destructors for dressings and refuse of a dangerous character is assuming increased importance. The "Horsfall" patent, the price of which should be well within the reach of the numerous small emergency hospitals now being organised, manufactured by the New Destructor Company, Limited, Atlas Works, Pershore, England, is well worth attention. The cupola type of "Horsfall" destructors is specially designed for such work, and is constructed for either forced or natural draught, and the No. 1 size is capable of destroying 20 cwt. a day with forced and 10 cwt. with chimney draught. A combustion chamber and flue for connection to the chimney are fitted, and, it should be remembered, it is semi-portable. Size No. 2 is of about half the capacity. Steel plate chimneys of any height can be supplied on request. Forced draught is supplied either by the manufacturers' steam blower or by an electric fan. Infectious hospitals have found this type of great value, and those organising emergency hospitals at the present time should make a point of remembering the need for a capable and at the same time convenient destructor of the "Horsfall" type.

